# Sirtuin 1 stimulates the proliferation and the expression of glycolysis genes in pancreatic neoplastic lesions

**DOI:** 10.18632/oncotarget.11013

**Published:** 2016-08-02

**Authors:** Andreia V. Pinho, Amanda Mawson, Anthony Gill, Mehreen Arshi, Max Warmerdam, Marc Giry-Laterriere, Nils Eling, Triyana Lie, Evelyne Kuster, Simone Camargo, Andrew V. Biankin, Jianmin Wu, Ilse Rooman

**Affiliations:** ^1^ Cancer Division, The Garvan Institute of Medical Research, Sydney, Australia; ^2^ St. Vincent's Clinical School, UNSW Australia, Sydney, Australia; ^3^ The Australian Pancreatic Cancer Genome Initiative, Darlinghurst, Australia; ^4^ University of Sydney, Sydney, Australia; ^5^ University of Zürich, Zürich, Switzerland; ^6^ Wolfson Wohl Cancer Research Centre, University of Glasgow, Glasgow, Scotland; ^7^ Center for Cancer Bioinformatics, Peking University Cancer Hospital and Institute, Beijing, China; ^8^ Oncology Research Centre, Vrije Universiteit Brussel, Brussels, Belgium

**Keywords:** pancreatic ductal adenocarcinoma, Sirtuin 1, tumorigenesis, proliferation, glycolysis

## Abstract

Metabolic reprogramming is a feature of neoplasia and tumor growth. Sirtuin 1 (SIRT1) is a lysine deacetylase of multiple targets including metabolic regulators such as p53. SIRT1 regulates metaplasia in the pancreas. Nevertheless, it is unclear if SIRT1 affects the development of neoplastic lesions and whether metabolic gene expression is altered.

To assess neoplastic lesion development, mice with a pancreas-specific loss of *Sirt1* (*Pdx1-Cre;Sirt1-lox*) were bred into a *Kras^G12D^* mutant background (*KC*) that predisposes to the development of pancreatic intra-epithelial neoplasia (PanIN) and ductal adenocarcinoma (PDAC). Similar grade PanIN lesions developed in *KC* and *KC;Sirt1-lox* mice but specifically early mucinous PanINs occupied 40% less area in the *KC;Sirt1-lox* line, attributed to reduced proliferation. This was accompanied by reduced expression of proteins in the glycolysis pathway, such as GLUT1 and GAPDH.

The stimulatory effect of SIRT1 on proliferation and glycolysis gene expression was confirmed in a human PDAC cell line. In resected PDAC samples, higher proliferation and expression of glycolysis genes correlated with poor patient survival. SIRT1 expression per se was not prognostic but low expression of Cell Cycle and Apoptosis Regulator 2 (CCAR2), a reported SIRT1 inhibitor, corresponded to poor patient survival.

These findings open perspectives for novel targeted therapies in pancreatic cancer.

## INTRODUCTION

The incidence of pancreatic cancer is on the rise, being projected to become the second cause of cancer-related deaths by 2030 [1]. Patients diagnosed with pancreatic ductal adenocarcinoma (PDAC), the most common form of pancreatic cancer, still face a very poor outcome with a 5-year survival of 5–7%. Surgical resection offers the best chance of cure; however most patients present with locally advanced or metastatic disease and are therefore ineligible for surgery [2]. Novel combination therapies such as nab-paclitaxel with gemcitabine or FOLFIRINOX have improved patient outcomes but improvements are small and toxicity is an issue [2].

Sirtuin1 (SIRT1) inhibitors have shown promising results in preclinical models of PDAC (reviewed in [3]). SIRT1 is an evolutionary conserved protein that senses NAD^+^ and resides in the nucleus or the cytoplasm where it can deacetylate a panel of proteins that regulate inflammation, lifespan and metabolic homeostasis, among many other processes (reviewed in [3–9]). The tumor suppressor p53 is the best studied protein that is deacetylated and inactivated by SIRT1 [10]. SIRT1 itself is regulated by Cell Cycle and Apoptosis Regulator 2 (CCAR2), an endogenous inhibitor of SIRT1's deacetylase activity in various organs, including in the pancreas [11].

Not only by its consumption of NAD^+^ does SIRT1 function as an energy sensor, it also mediates metabolic effects in liver, skeletal muscle, heart and adipocytes by directly deacetylating proteins that regulate gluconeogenesis, fatty acid metabolism and glycolysis [12]. Through effects on Phosphoglycerate 1 (PGAM1) and peroxisome proliferator-activated receptor alpha coactivator 1 alpha (PGC1a), SIRT1 was found a negative regulator of glycolysis in liver and skeletal muscle [12]. Contrary, through inactivation of p53 SIRT1 can stimulate glycolysis [13], underscoring the dependency on the context.

The biological functions of SIRT1 in PDAC are still poorly understood. We recently reported a role for SIRT1 in the development of pancreatic acinar-ductal metaplasia, a process that occurs in acute and chronic pancreatitis and may give rise to neoplastic lesions [14]. We now sought to explore if SIRT1 also impacts the development of (pre)tumoral lesions. In addition, we analyzed a potential metabolic regulation by SIRT1 in pancreatic lesions.

## RESULTS

### Sirt1 promotes the proliferation of mucinous intraepithelial neoplastic lesions in the murine pancreas

**Figure 1:**
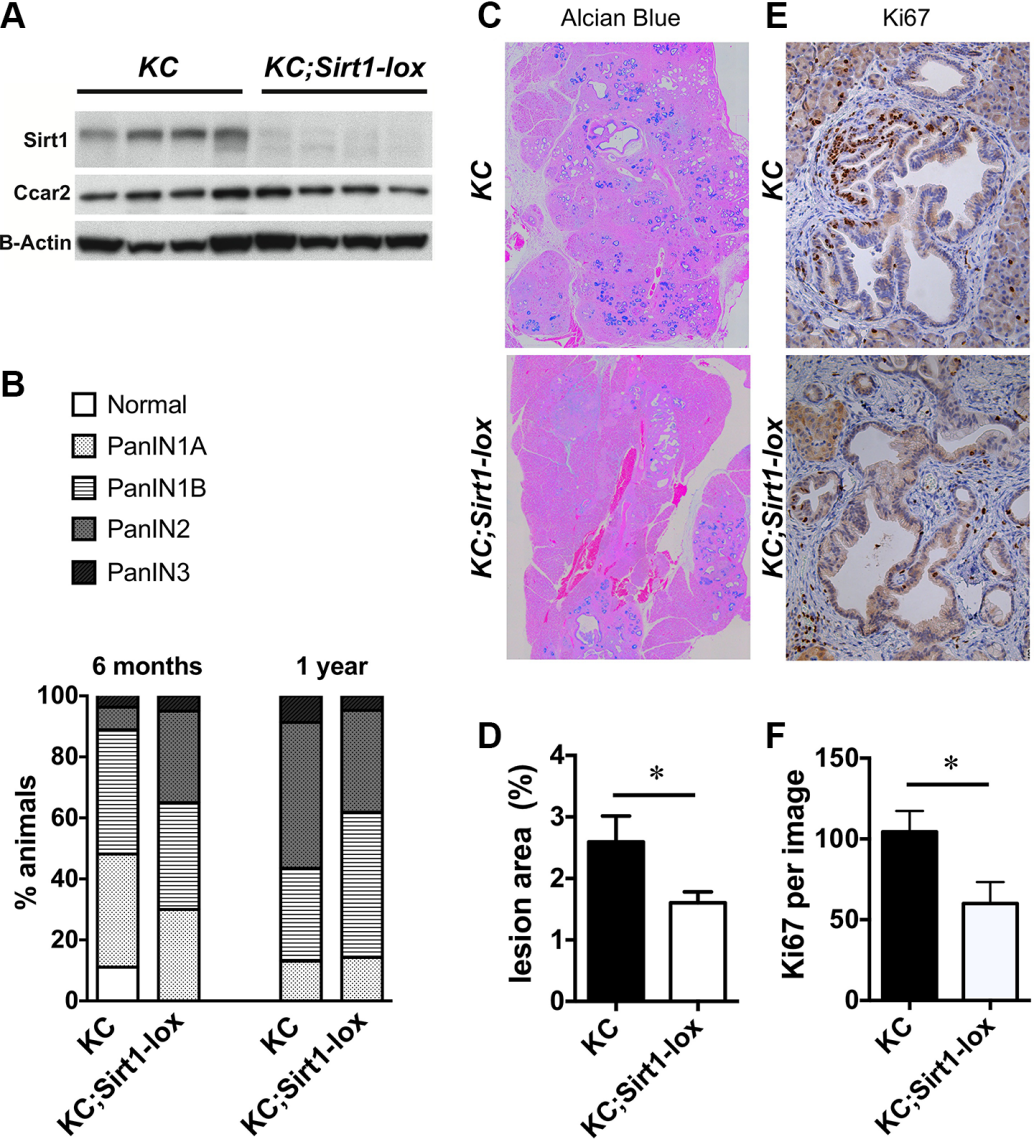
Pancreatic lesions in *KC* and homozygous *KC;Sirt1-lox* mice (**A**) Western Blot analysis of Sirt1, Ccar2 and Beta-Actin (B-Actin) protein expression in whole pancreas tissue, *n* = 4 per group. (**B**) Percentage of animals with the highest lesion in pancreas tissue sections that were classified as normal, PanIN 1,2 or 3. *n* = 20 *KC;Sirt1-lox* and *n* = 27 *KC* at 6 months of age, *n* = 21 *KC;Sirt1-lox* and *n* = 23 *KC* at 12 months of age. (**C**) Representative Alcian Blue staining in pancreas tissue sections of 12 month old animals. (**D**) Measurement of the percentage Alcian Blue positive area in pancreas tissue sections. *n* = 9–11. (**E**) Representative Ki67 immunohistochemistry in pancreas tissue sections of 12 month old animals. (**F**) Quantification of Ki67 positivity per image of lesion area. *n* = 9–13. All data are represented as mean +/− SEM, *P < 0.05.

Mice with an activating mutation in *Kras* (*lox-STOP-lox Kras*^G12D^) [15] that is specifically targeted to the pancreas by a *Pdx1-Cre* driver (abbreviated *KC* mice) were crossed with a mouse line in which the exon 4 of *Sirt1* is flanked by loxP sites to obtain homozygous Sirt1-deficient KC mice (*KC;Sirt1-lox*) [14, 16]. Only very low expression of the mutant form of SIRT1 protein is found in the pancreas of the *KC;Sirt1-lox* animals (Figure 1A and [16]). Cheng et al. have previously shown that the resulting short mutant form of SIRT1 lacks SIRT1 deacetylation activity, resulting in a phenotype similar to a full SIRT1 knock out [17]. *KC;Sirt1-lox* animals were sacrificed at 6 and 12 months of age, in parallel with a cohort of *KC* control mice. We observed that in the mice that had not been culled earlier because of reaching ethical endpoints (due to PDAC, another tumor or unknown cause), PanIN1-3 lesions progressively developed at 6 and 12 months of age (Figure 1B). The progression of the lesions scored by the grade of the PanINs did not differ when comparing *KC* controls with *KC;Sirt1-lox* mice (Figure 1B).

To evaluate the lesion burden, we quantified the area occupied by PanIN lesions using two different methods: Keratin 19 (Krt19) immunohistochemistry and Alcian Blue staining of mucinous lesions. The relative area of Krt19 positive lesions presented no differences between *KC* controls and *KC;Sirt1-lox* mice (Supplementary Figure S1). However, the area of mucinous early PanINs, measured by the percentage of Alcian Blue–positive, was significantly lower in the *KC;Sirt1-lox* mice (1.6 ± 0.2% versus 2.6 ± 0.4% area, *n* = 9–11, *P <* 0.05, Figure 1C, 1D).

The reduced area occupied by mucinous neoplastic lesions was attributed to lower proliferation, measured by Ki67 immunohistochemistry (IHC) in the *KC;Sirt1-lox* background compared to *KC* controls (60 ± 13 versus 104 ± 13 counts of Ki67 positive cells per field, *n* = 9–13, *P <* 0.05, Figure 1E, 1F). There was no statistically significant difference in the proliferation of the neighboring stroma. In addition, all mice analyzed only showed very low apoptosis in the lesions, detected by cleaved caspase-3 staining (not shown). Pancreata from *Kras* wild type *Pdx1-Cre;Sirt1-lox* mice did not show alterations in the proliferation of any cell type (acinar, duct, islets or stroma) when compared with *Pdx1-Cre* controls (Supplementary Figure S2), suggesting the effect on proliferation is specific for the early lesions in the *Kras* mutant background.

In conclusion, SIRT1 is dispensable for pancreatic lesion development, does not affect the proliferation of normal pancreatic tissue but specifically stimulates the growth of early mucinous PanIN lesions.

### SIRT1 regulates the expression of glycolysis genes in mouse pancreas

Ample evidence is available that proliferation of (pancreatic) tumor cells is enabled by an increased rate of intracellular glucose import and a higher rate of aerobic glycolysis (referred to as the Warburg effect) [18]. Moreover, SIRT1 is a known metabolic regulator and deacetylates several proteins that play critical roles in glycolysis such as p53 [13] and MYC [19]. Therefore, we verified if SIRT1's effect on proliferation of the pancreatic lesions involved changes in glycolysis genes.

**Figure 2:**
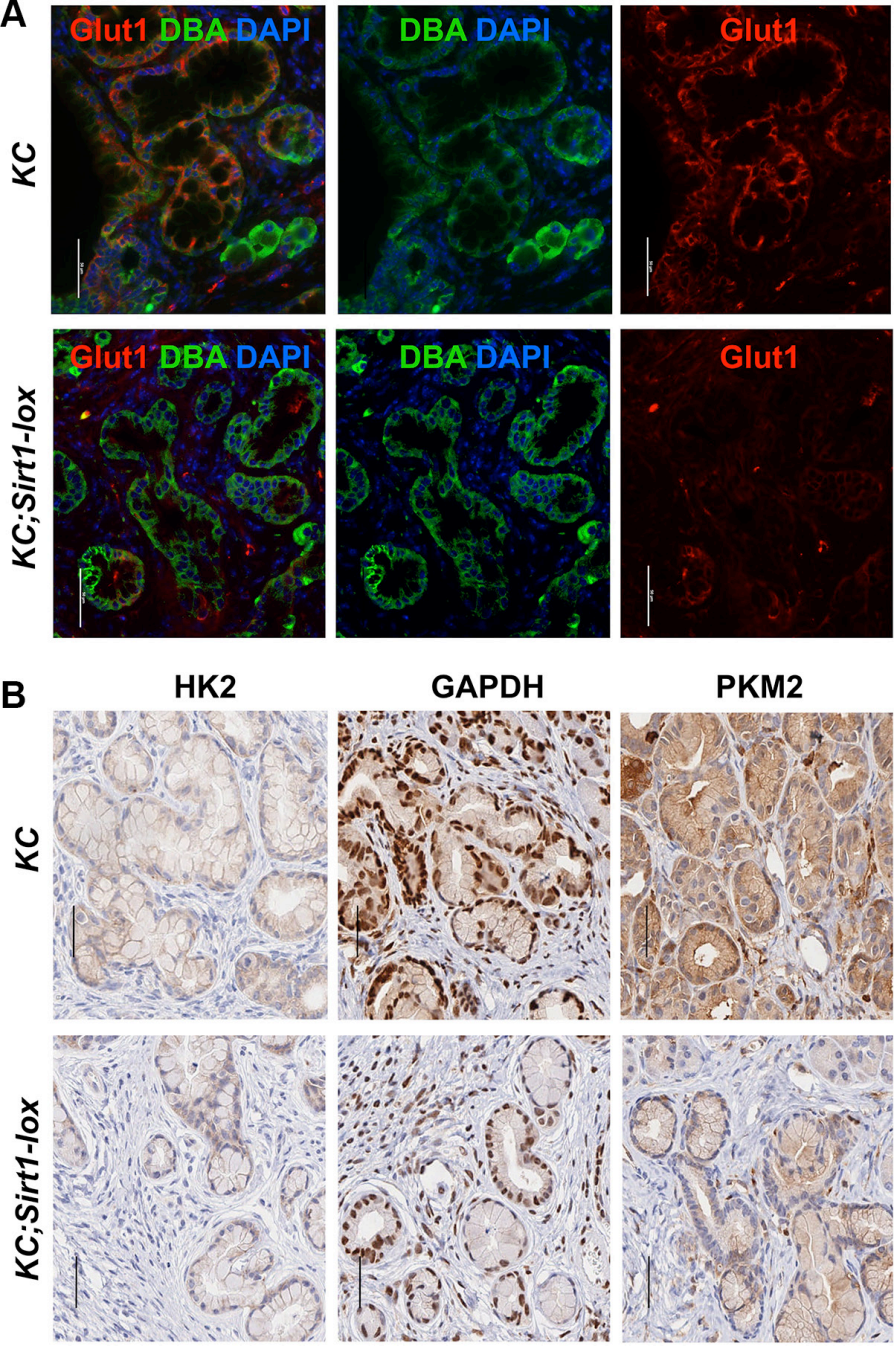
Expression of glycolytic proteins in murine SIRT1-deficient PanIN lesions (**A**) Immunofluorescence for GLUT1 (red) and (**B**) immunohistochemistry for HK2, GAPDH and PKM2 in pancreas tissue sections (bars = 50 micrometer).

We used our SIRT1-deficient mouse model to verify a regulation of glycolysis proteins by SIRT1 in pancreatic lesions *in vivo.* When comparing immunostainings in the PanIN lesions in *KC;Sirt1-lox* versus *KC* controls, we found a weaker staining for GLUT1 (Glucose transporter 1), GAPDH (Glyceraldehyde 3-phosphate dehydrogenase) and PKM2 (Pyruvate kinase isozyme m2) and slightly weaker HK2 (Hexokinase 2) (Figure 2).

In conclusion, SIRT1 stimulates the expression of glycolysis genes in mouse PanIN lesions.

### SIRT1 stimulates proliferation and glycolysis gene expression in human PDAC

**Figure 3:**
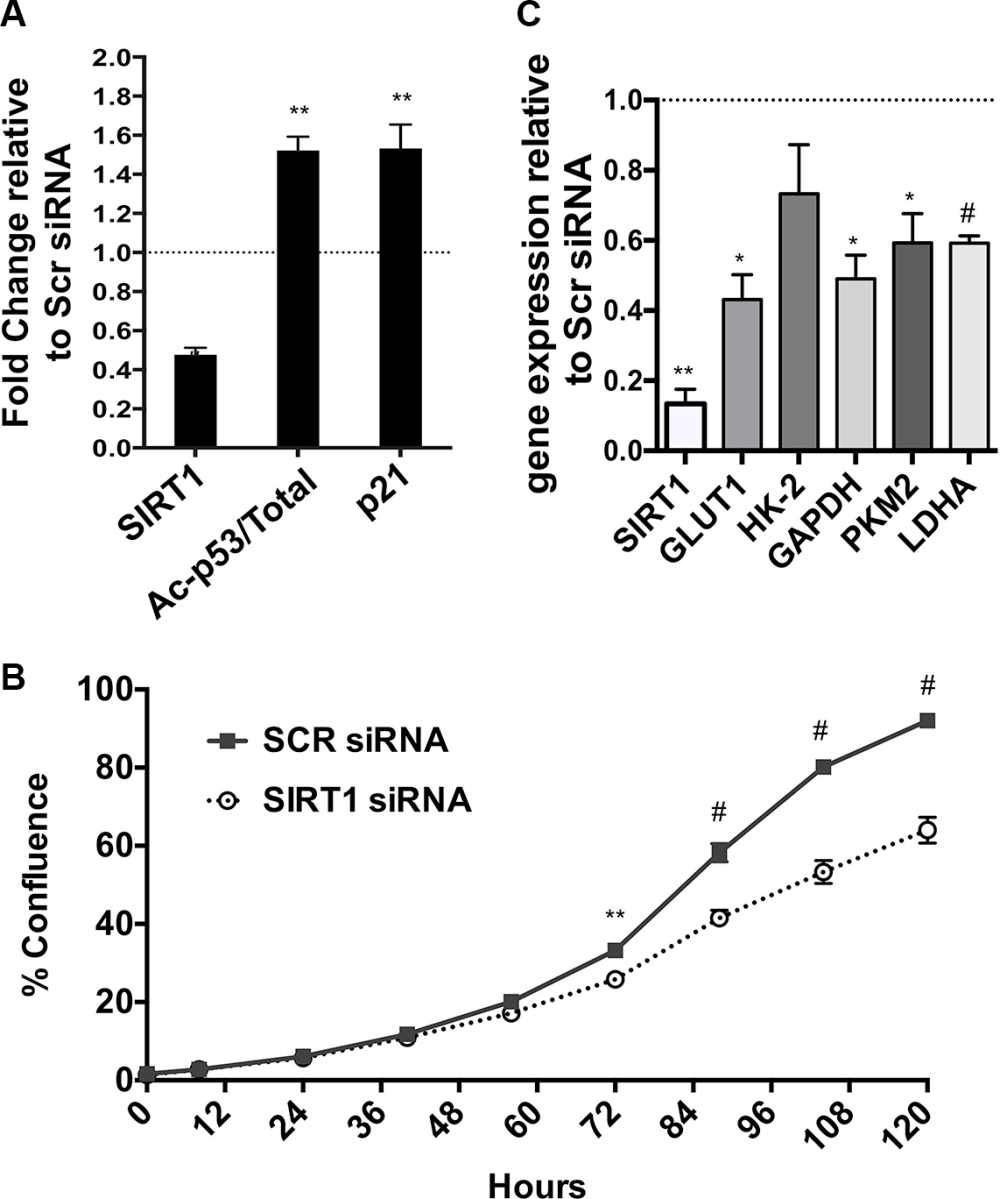
Effects of SIRT1 siRNA in human PDAC cells (**A**) Band density quantification by Image J analysis of Western Blot of SIRT1, acetylated and total p53 and p21, using B-ACTIN as a loading control, from Panc1 cells treated with a scrambled siRNA sequence or with siRNA for SIRT1. *n* = 6 (See representative Western Blot in Supplementary. Figure S3). (**B**) Real time RT-PCR analysis of the mRNA expression of SIRT1, GLUT1, HK2, GAPDH, PKM2, LDHA and PDK1 relative to HPRT as housekeeping gene (*n* = 4) in SIRT1 siRNA treated Panc1 cells. (**C**) IncuCyte graph of the % increase in cell confluence in siRNA treated cells versus scr controls. One of three representative experiments is shown. All data are represented as mean +/− SEM, *P < 0.05, **P < 0.01, ^#^*P* < 0.001.

Complementary to our *in vivo* observations in the mice, we down-regulated SIRT1 gene expression using siRNA in a human pancreatic tumor cell line. Effective knock down of SIRT1 in Panc1 cells was demonstrated by reduced SIRT1 protein in Western Blotting analysis, along with increased acetylation of SIRT1's best-characterized substrate p53 [10] and downstream upregulation of p21, a known p53 target gene (Figure 3A and Supplementary Figure S3). We found that knock down of SIRT1 impaired the proliferation of the PDAC cells, an extension of previous reports [14, 20] (Figure 3B). The cells treated with siRNA for SIRT1 had a consistent reduction in the mRNA of SIRT1, GLUT1 (Glucose transporter 1), GAPDH, PKM2 and LDHA (Lactate dehydrogenase A) (Figure 3C).

### High proliferation, high glycolysis gene expression and low expression of a SIRT1 inhibitor corresponds to the poorest patient survival outcomes

**Figure 4:**
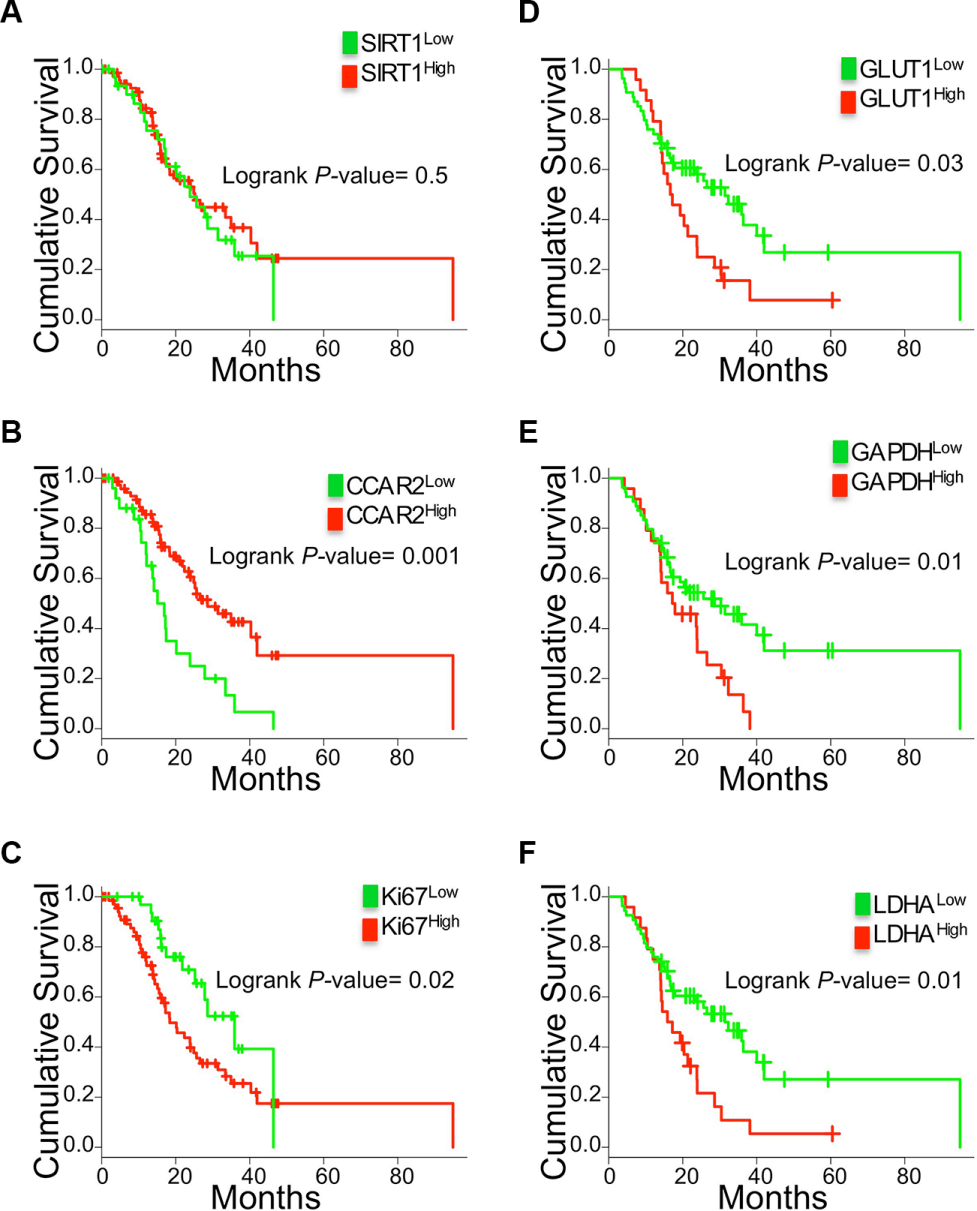
Correlation of SIRT1, CCAR2, Ki67 and glycolysis genes with PDAC patient survival outcomes Kaplan Meier curves for the protein expression of SIRT1 (**A**) and CCAR2 (**B**) and Ki67 (**C**) scored in the tumor epithelium of PDAC patients (*n* = 104). Kaplan Meier curves of GLUT1 (**D**), GAPDH (**E**) and LDHA (**F**) gene expression in high cellularity patient samples (21) (*n* = 78).

We analyzed if SIRT1 expression was prognostic in human PDAC, using a patient cohort of treatment-naive early stage I and II resected PDAC (*n* = 104) [21]. Staining for SIRT1 protein expression (nuclear, cytoplasmic or total) in the PDAC cells, following the protocol we had previously optimized in another smaller cohort [14], did not directly correlate with survival outcome (Figure 4A and not shown). We verified if instead, expression of the SIRT1 inhibitor CCAR2 was related to clinical outcomes. CCAR2 protein expression is very high in the majority of the samples (H-score > 200, Supplementary Figure S4) and correlated with improved survival; the 25% low CCAR2 expressing tumors corresponded to patients with poorer survival (Figure 4B). The low CCAR2 PDAC also independently correlated with undifferentiated and poorly differentiated tumors (*n* = 104, *P <* 0.01). Pathway analysis of the 88 genes that positively correlated with CCAR2 gene expression in microarray data of these human PDAC samples [21] resulted in ‘chromatin modification’, ‘histone acetylation’ and ‘cell cycle’ (Table 1, *n* = 104), supportive of a regulatory role of CCAR2 in typical SIRT1 activities in PDAC.

**Table 1:** Reactome pathways enriched among the genes that positively correlated (Pearson and Spermann correlation coefficient ≥ 0.4) with CCAR2 in cDNA microarray analysis of PDAC (*n* = 104)

#Term	Corrected *P*-Value
Chromatin modifying enzymes	0, 0008
Chromatin organization	0, 0008
RNA transport	0, 0014
HATs acetylate histones	0, 0030
mRNA surveillance pathway	0, 0056
G2/M Transition	0, 0069
Mitotic G2-G2/M phases	0, 0069
Centrosome maturation	0, 0082
Recruitment of mitotic centrosome proteins and complexes	0, 0082

Then we assessed the clinical significance of increased epithelial proliferation and glycolysis gene expression, the latter confined to high cellularity samples (*n* = 78) to enrich for the epithelial gene expression. Higher percentage of Ki67-stained epithelium correlated with poor outcomes (Figure 4C). Also high SLC2A1/GLUT1, GAPDH and LDHA but not PKM2 correlated with worse patient survival (Figure 4D–4F and not shown).

In conclusion, CCAR2 protein but not SIRT1 is prognostic in PDAC. In addition, reduced patient survival time is seen in tumors that proliferate more and have higher expression of glycolysis genes.

## DISCUSSION

SIRT1 has been attributed roles in tumor formation [7, 22–24] and is a regulator of metabolism, a process that interconnects with cancer [4, 25]. Previously we had shown a role of SIRT1 in metaplasia of pancreatic cells [14, 16]. Because of the link between glycolytic metabolism, tumor formation and tumor cell growth, we set out to explore whether these mechanisms were regulated by SIRT1 in the pancreas.

The development of pancreatic lesions is dependent on the activation of KRAS [15, 26, 27]. In the vast majority of PDAC patients, the KRAS gene presents an activating mutation [21]. Here we investigated the effects of SIRT1 inactivation in a *KRAS* mutant background using genetically engineered mice [15]. We are the first to demonstrate that a pancreas-specific inactivation of SIRT1 limited the proliferation of early mucinous PanIN lesions. Similar to our observations, SIRT1-deficiency reduced polyp area and size in the APC+/min model of colorectal cancer [23] and reduced the number and the size of tumors in a colitis-induced colorectal cancer model [24]. We extended our findings on tumor development to tumor progression, where we also found that SIRT1 stimulated the proliferation of established pancreatic tumor cells, in line with previous reports (reviewed in [3]).

Aerobic glycolysis plays a central role in cellular transformation and the proliferation of tumors [18]. In PDAC, aerobic glycolysis is a source of intermediates for the non-oxidative pentose phosphate pathway [30] [31]. Our analyses demonstrated that SIRT1 positively regulated the expression of key genes in the glycolysis pathway. This new finding was made, both in the *Kras* mutant mouse model and in *KRAS* mutant human PDAC cells. A recent paper by Daemen *et al* [32] found that pancreatic tumor cell lines can be sub-typed according to their gene expression signature and metabolic profile with the quasi-mesenchymal sub-type having a glycolytic nature. Our patient samples were categorized by this gene expression signature into exocrine, quasi-mesenchymal and classical subtype (as also reported in [33]) but did not show sub-type specific differences in SIRT1 expression (not shown).

Several genes may be mediating the SIRT1 dependent regulation of glycolytic gene expression; SIRT1 deacetylates and inactivates p53 which is a negatively regulator of glycolysis [13]. We detected consistent changes in acetylation of p53 upon manipulation of SIRT1 in a PDAC cell line. Despite our attempts, we did not succeed in detecting acetylated P53 in tissue sections of human PDAC. More in-depth study and suitable antibodies would be required to decipher if indeed p53 is the critical mediator. Other genes may be accountable such as MYC, a transcriptional activator of glycolysis genes, since SIRT1 is reported to increase MYC's transcriptional program [19, 22]. Alternatively, SIRT1 protects HIF1a from acetylation and degradation and thereby can increase GLUT1 expression, a mechanism reported before [34]. Although not specific for SIRT1, HDAC inhibitors were found to down-regulate GLUT1 expression and to inhibit hexokinase 1 enzymatic activity in multiple myeloma cells [35], underscoring that acetylation plays a key role in regulation of glycolytic metabolism. The opposite regulation has also been reported, e.g. inhibition of SIRT1 leads to acetylation and degradation of PKM2 while stimulating tumor proliferation [36]. Apart from glycolysis, other energy generating and biosynthetic pathways need to be controlled by the tumor cells in order to promote their cell proliferation [2–4]. SIRT1 deacetylates and thereby stimulates PGC1α. PGC1α is a negative regulator of glycolysis but promotes tumor progression through oxidative phosphorylation and lipogenesis [37] and by increasing mitochondrial gene expression that protects against pancreatic tumour cell apoptosis [38]. Therefore, SIRT1's effect on PGC1α may also have contributed to our observations. We note that SIRT1 was reported to deacetylate KRAS thereby promoting its downstream signaling [39]. Knowing that oncogenic KRAS signaling upregulates many key enzymes in glycolysis (extensively reviewed in [40]), this may be another mechanism by which SIRT1 indirectly promotes glycolysis.

Despite the difference in area occupied by mucinous Alcian Blue positive lesions and the reduced expression of proteins in the glycolytic pathway, total area of lesions, as stained by Krt19 showed no differences between controls and SIRT1 deficient mice. Our observation suggests that only the early PanIN stage is affected in the SIRT1 loss of function model. We speculate that a subset of cells have started to compensate for the lower proliferation/reduced glycolysis of the early PanIN lesions. These cells must have progressed faster or have skipped the early PanIN stage. It has been reported that pancreatic tumor cells, upon genetic or pharmacological ablation of the bulk of glycolysis-dependent cells can become more aggressive and can lead to full recurrence of the tumor. This involves the acquisition of stem cell features and metabolic reprogramming with a shift from glycolysis to oxidative phosphorylation and is notably dependent on PCG1a [28], a protein well known for its regulation by SIRT1 [29], and important in tumor growth, as outlined above.

We verified the significance of our findings in clinical samples. In a cohort of human early stage tumors, high proliferation and high expression of glycolysis genes corresponded, not unexpectedly, to poorer patient outcomes. This inferred that high expression of SIRT1 might also lead to worse outcome. However, expression of SIRT1 in our analysis did not directly correlate with the patient survival outcomes, in discrepancy with other studies reporting that SIRT1 in PDAC correlated with poor histological differentiation and poor postsurgical survival [41] and with increased size, lymph node burden and hepatic metastasis [20]. Nevertheless, we found that CCAR2, an inhibitor of SIRT1's deacetylase activity, was prognostic in our cohort, with the lower CCAR2 expressing tumors corresponding to poorly differentiated high mortality tumors. Based on literature [11, 42], the lowest CCAR2 expressing tumors would have the highest SIRT1 activity. Microarray data analysis of the patient samples also further suggested that CCAR2 regulates typical SIRT1 activities such as histone deacetylation but more research and the development of suitable antibodies would be needed before CCAR2 could be definitely used as a surrogate marker of low SIRT1 activity. Indeed, CCAR2 may also exert other effects independent of SIRT1 inhibition; for example, a recent report showed that CCAR2 is a tumor suppressor that stabilizes p53 with effects independent of SIRT1 [43].

In conclusion, our new results underscore a novel oncogenic function of SIRT1 during pancreatic cancer development where it stimulates the proliferation and expression of glycolytic proteins. SIRT1 is an interesting druggable target to halt tumor cell proliferation with successes in chronic myeloid leukemia [44] and with an inhibitor found to be safe in a clinical trial [45, 46]. Several groups have found promising effects of SIRT1 drugs in preclinical models of PDAC [3]. Our data do not only reinforce the idea to explore SIRT1 as a druggable target in early stage pancreatic cancer, we also deciphered a relation with tumor metabolism. We found that SIRT1 is a stimulus for glycolytic enzymes. Interestingly, specific drugs for these glycolytic enzymes are currently being tested, e.g. the LDH-A antagonist FX11 in PDAC [47] or the Hexokinase II inhibitor, 3-BrPA in breast cancer [48]. One could envision a combination therapy of a SIRT1 inhibitor and one of these drugs that target the metabolism of the pancreatic tumor or one could potentially use low CCAR2 expression, likely corresponding to high SIRT1 activity, as a biomarker to select a population of early stage PDAC patients for therapy with drugs that target the glycolytic metabolism. Our study paves the way for further investigation into these new therapeutic angles.

## MATERIALS AND METHODS

### Animals and *in vivo* experimentation

B6;129-*Sirt1*^tm1Ygu^/J (*Sirt1-lox*) strains were obtained from Jackson Laboratories and bred with the B6.FVB-*Tg(Ipf1-cre)6Tuv*/J (*Pdx1-Cre*, abbreviated as C) [15] and the *lox-STOP-lox-Kras*^G12D^ line (abbreviated as *KC*) [15] to create *KC;Sirt1-lox* mice. The *Sirt1-lox* mice [49] have a loxP-flanked neomycin cassette just upstream of exon 4, and a third loxP site just downstream of exon 4 (encoding a conserved *Sirt1* motif) of the targeted gene. The resulting offspring have exon 4 deleted in the pancreas. In all experiments, only homozygous *Sirt1-lox* mice were analyzed and the respective *Sirt1* wild-type line was used as control. All animal experiments were approved by the Garvan Animal Ethical Committee (AEC approval #12-52).

### Patient derived tissue collection

Biospecimens (tissue microarrays of paraffin embedded material and microarray gene expression datasets) and the clinico-pathological data were provided by the Australian Pancreatic Cancer Genome Initiative (APGI) [21, 50, 51] (ethical approval:HREC/11/RPAH/329 – The Molecular Pathology of Pancreatic Cancer, incorporating the APGI, approved by Sydney Local Health District –RPA Zone, protocol x11-0220).

### Immunostaining

Mouse pancreas tissues were fixed in 4% formalin and embedded in paraffin. All immunohistochemistry was performed using the Dako Autostainer, Universal Staining System Model# LV-1 and the Leica BOND RX, according to supplier procedures. After deparaffinization and antigen retrieval (S1699 Target Retrieval Solution, Dako), slides were incubated with diluted primary antibody (Supplementary. Table S1) for 60 minutes and a secondary EnVision antibody (Dako) (Supplementary. Table S1) for 30 minutes. Finally the slides were treated with 3,3′-Diaminobenzidine (DAB+ Substrate Chrmogen system, Dako) for 10 minutes and counterstained with haematoxylin. All immunohistochemistry images were taken using the Aperio Scanscope CS system (Leica Biosystems). For immunofluorescence of GLUT-1, mouse pancreas was treated with EDTA-NaOH antigen retrieval (boiling 20 minutes in 1 mM pH 8). Sections were incubated with primary antibody overnight at 4°C and subsequently with anti-mouse-594, DBA and Dapi. Sections were viewed on a Nikon Eclipse TE300 epifluorescence microscope (Nikon Instruments Inc, Melville, NY) equipped with a DS-5M Standard charge-coupled device camera (Nikon Instruments Inc) and acquired with NISElements (Nikon Instruments Inc).

Human PDAC samples were assembled by APGI into tissue microarrays containing 3 samples of each patient tumor. SIRT1 and CCAR2 immunohistochemistry (IHC) on the human tissue microarrays was performed as reported before [14] and was scored blindly as absolute intensity (arbitrary values 0–3) and an estimated percentage of positive nuclei (H-score).

### Alcian blue staining

Tissue slides were manually stained with Clinipure ALCBLUE1 03 (1% alcian blue, 3% acetic acid solution) for 30 minutes and counterstained with eosin. Alcian Blue quantification was performed by analyzing the stained slides with the Leica DM6000 Power Mosaic microscope and software. Twenty-five pictures per sample were manually selected to discard any images with empty areas or non-pancreatic tissue like lymphoid tissue, blood vessels or fat. The stained area was measured and the percentage of stained area over total tissue area was calculated.

### siRNA knockdown of SIRT1

Panc-1 cells were obtained from the American Type Culture Collection (ATCC) Cell Biology Collection and used within 6 months of revival. The cells were tested for Mycoplasma contamination. The cells were plated in DMEM +10% Fetal Bovine Serum. At 24 hours, cells were transfected with 10 nM siRNA / Hyperfect complexes according to the manufacturers instructions (Qiagen) and incubated 37C, 5% CO2. One of two successful siRNAs (Invitrogen) was used for further analysis. Panc1 seeded in 96 well plates at 2 × 10^3^ cells were monitored for growth (percent confluence) for up to 5 days following siRNA treatment using the IncuCyteZOOM live cell imager (Essen Bioscience). Replicate plates were also analysed for cell proliferation via BRDU ELISA (Roche, 11647229001) with a 2 hour Bromodeoxyuridine pulse at 96 hours per manufacturers instructions.

### Western blotting

Cells were lysed in Normal Lysis Buffer (Hepes 50 mM, NaCl 150 mM, Glycerol 10%, Triton X100 1%, MgCl2 1.5 mM, EGTA 1 mM, Pyrophosphate 10 mM, NaF 100 mM) complemented with protease and phosphatase inhibitors (Roche) and MG132 (10 ug/ml). Frozen tissue specimens were homogenized in RIPA Buffer (Tris-Hcl 50 mM, NaCl 150 mM, EDTA 5 mM, NP40 0.5%, 0.1%, SDS 0.1%) complemented with protease and phosphatase inhibitors and MG132. Protein extracts were subjected to electrophoresis on 4–12% gradient polyacrylamide gels (SDS-PAGE) and transferred onto a PVDF membrane (Biorad). The membrane was incubated overnight at 4°C with primary antibodies (Supplementary. Table S1) diluted in TBS/BSA (Tris pH 7.4 10 mM, NaCl 150 mM, 5% BSA, 0.05% Sodium Azide and phenol red). The membrane was then incubated with HRP-conjugated secondary antibodies at a 1/2000 dilution. The signals were visualized with ECL (Perkin Elmer) and autoradiography, and band densities of the gene of interest relative to the housekeeping gene were measured with ImageJ software.

### Real time PCR analysis

Total RNA from Panc1 cells was isolated using the Purelink RNA mini kit (Ambion, Life Technologies) and subjected to DNase1 treatment (Ambion, Life Technologies). First strand cDNA synthesis was performed with a random hexamer / poly-A primer mix (Roche) according to manufacturers instructions. Real time PCR products were amplified with Fast Start SYBR Green (Roche) using the primers listed in Supplementary. Table S2 using the 7900HT Fast Real Time PCR System (Applied Biosystems). PCR product specificity was confirmed with dissociation curve analysis and 2% agarose gel electrophoresis of products. Expression levels were normalized to house keeping gene expression (*HPRT*).

### Statistics

Results are presented as mean ± SEM. The number of independent experiments is represented as n. Data were analyzed by Prism 6.0 using Student *t* test (unpaired *t* test) or two-way ANOVA and results considered significant when *P* < 0.05.

## SUPPLEMENTARY MATERIALS FIGURES AND TABLES


