# Supplementary material for: Sirtuin 1 stimulates the proliferation and the expression of glycolysis genes in pancreatic neoplastic lesions
Source: Oncotarget. 2016 Aug 2;7(46):74768–78. doi: 10.18632/oncotarget.11013 (PMC5342700; doi:10.18632/oncotarget.11013)
Supplement: Supplementary file 1 [file oncotarget-07-74768-s001.pdf]

Sirtuin 1 stimulates the proliferation and the expression of glycolysis genes in pancreatic neoplastic lesions

Supplementary Materials

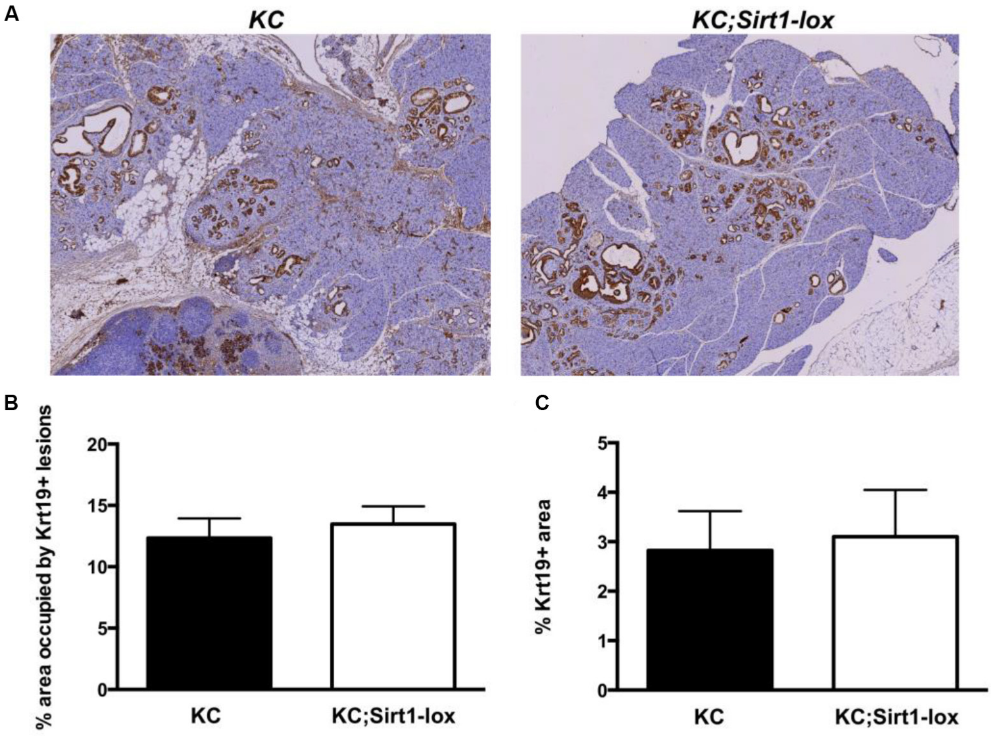

Supplementary Figure S1: Krt19 immunohistochemistry in 12 month old *KC* and homozygous *KC;Sirt1-lox* mice. (**A**) Representative images of Krt19 immunohistochemistry in *KC* and *KC;Sirt1-lox* mice pancreata. (**B**) Manual quantification of area occupied by Krt19+ lesions (includes lumen of lesions). (**C**) Automated quantification of Krt19+ area (excluding lumen of lesions). Bar = 100 µm.

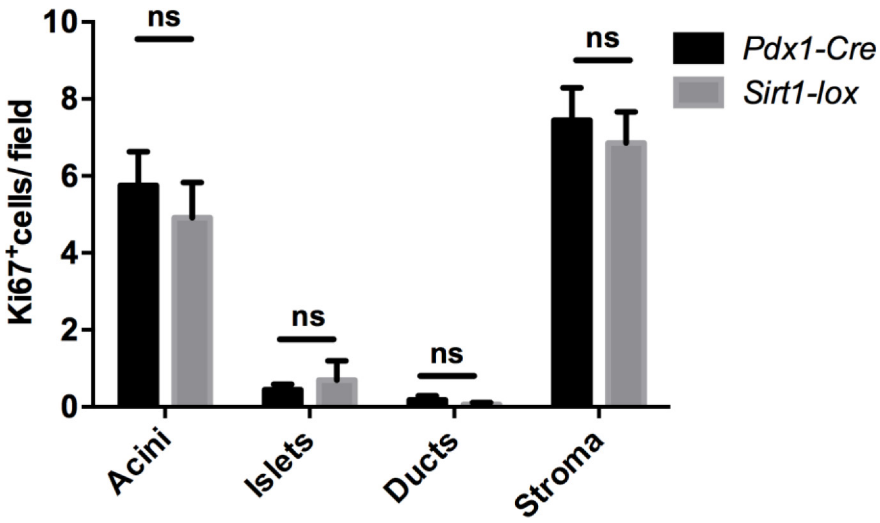

Supplementary Figure S2: Cell proliferation in pancreata of normal and *Sirt1-lox* animals. Quantification of Ki67+ cells in *Pdx1-Cre* and *Sirt1-lox* mice pancreata

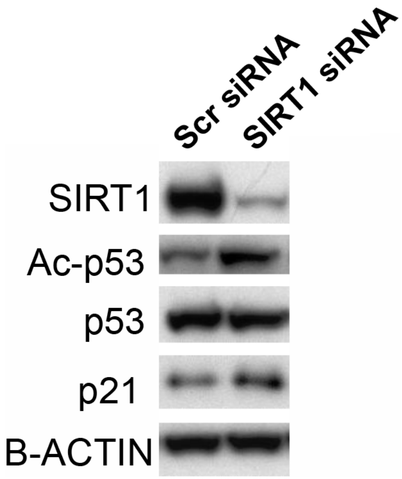

Supplementary Figure S3: Effects of SIRT1 siRNA in human PDAC cells. (**A**) Representative Western Blot of SIRT1, acetylated and total p53, p21 and B-ACTIN as a loading control from Panc1 cells treated with a scrambled siRNA sequence or with siRNA for SIRT1.

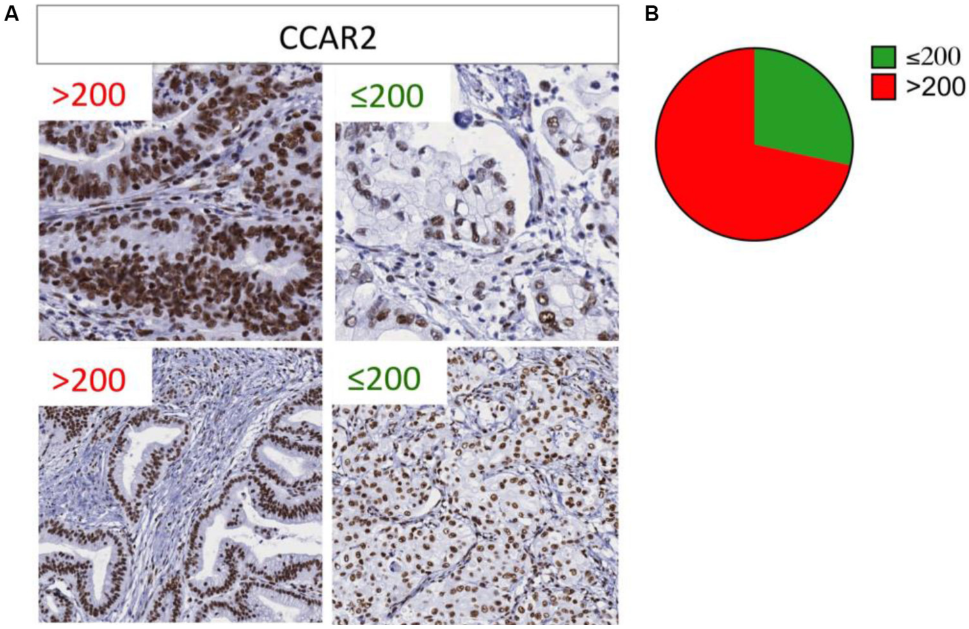

Supplementary Figure S4: CCAR2 expression in human PDAC samples. (**A**) Representative images of CCAR2 immunohistochemistry in groups that have an H-score of > 200 and ≤ 200. B) Pie chart representing the groups according to H-score > 200 and ≤ 200 (n = 104).

Supplementary Table S1: List of primary and secondary antibodies, used in immunostainings and Western Blot analysis

Primary antibodies

Antigen	Host	Type	Manufacturer
Sirtuin 1	Rabbit	Polyclonal	Sigma (HPA006295)
CCAR2	Rabbit	Polyclonal	Sigma (HPA019943)
Acetyl-p53 (Lys373, Lys382)	Rabbit	Polyclonal	Millipore (06-758)
p53	Mouse	Monoclonal	Santa-Cruz (SC-126)
Krt19	Rat	Monoclonal	Developmental Stud ies Hybridoma Bank (TROMA-III)
Glut1	Mouse	Monoclonal	Abcam (ab40084)
Hexokinase II	Rabbit	Monoclonal	Cell Signaling (2867)
Gapdh	Rabbit	Monoclonal	Cell Signaling (5174)
Pkm2	Rabbit	Monoclonal	Cell Signaling (4053)
Beta-actin	Mouse	Monoclonal	Sigma (AC5441)
DBA-FITC	–	–	Vector Laboratories (FL-1031)

Secondary antibodies

Anti	Host	Label	Manufacturer
Mouse	Donkey	Alexa Fluor® 594	Thermo Fisher (R37115)
Rabbit	Donkey	Horseradish peroxidase	GE Healthcare (NA934)
Mouse	Sheep	Horseradish peroxidase	GE Healthcare (NA931)

Supplementary Table S2: List of forward and reverse primer sequences used for RT-qPCR analysis

qPCR primers

Gene	Forward primer	Reverse primer
HPRT	GGCTCCGTTATGGCGACCC	TGTGATGGCCTCCCATCTCCTT
SIRT1	TTGCAACAGCATCTTGCCTG	GTTCATCAGCTGGGCACCTA
CCAR2	TCTTCCAAACATCCCACACAC	GCCCGCTGTTTGCGTTTC
GLUT1	CCTGCAGTTTGGCTACAACAC	CAGGATGCTCTCCCCATAGC
HK2	CCCTGAGGACATCATGCGAG	GGTCCATGAGACCAGGAAACT
GAPDH	TGCACCACCAACTGCTTAGC	GGCATGGACTGTGGTCATGAG
PKM2	GTGGCTCGTGGTGATCTA GG	CCAGACTTGGTGAGGACGAT
LDHA	TGGATCTCCAACATGGCAGC	CCAAGCCACGTAGGTCAAGA
PDK1	GCAAAGTTGGTATATCCAAAGCC	GTACATTGCAGTTTGGATTTATGC
PDHA1	CGAGAGGCAACAAGGTTTGC	TCAAAAGGTGGGTCGCTGGA
